# Improving the diagnostic of absorptive hypercalciuria: a comparative analysis of calcium load tests at 2-hour and 4-hour intervals

**DOI:** 10.1093/ckj/sfae399

**Published:** 2024-12-09

**Authors:** Lara Cabezas, Pierre Letourneau, Aurélie De Mul, Justine Bacchetta, Laurence Chardon, Laurence Derain Dubourg, Sandrine Lemoine

**Affiliations:** Service de Néphrologie, HTA, dialyse et explorations fonctionnelles rénales, Hopital Edouard Herriot, Hospices civils de Lyon, Lyon, France; Nephrology, Hemodialysis, Apheresis and Kidney Transplantation Department, University Hospital Grenoble, Grenoble, France; Service de Néphrologie, HTA, dialyse et explorations fonctionnelles rénales, Hopital Edouard Herriot, Hospices civils de Lyon, Lyon, France; Service de Néphrologie, HTA, dialyse et explorations fonctionnelles rénales, Hopital Edouard Herriot, Hospices civils de Lyon, Lyon, France; INSERM 1060-CARMEN, Université de Lyon, Université Lyon 1, Lyon, France; Centre de Référence des Maladies Rénales Rares, Centre de Référence des Maladies Rares du Calcium et du Phosphore, HFME, Bron, France; Hospices civils de Lyon, Groupement hospitalier Est, Service de biochimie, Lyon, France; Service de Néphrologie, HTA, dialyse et explorations fonctionnelles rénales, Hopital Edouard Herriot, Hospices civils de Lyon, Lyon, France; Centre de Référence des Maladies Rénales Rares, Centre de Référence des Maladies Rares du Calcium et du Phosphore, HFME, Bron, France; Service de Néphrologie, HTA, dialyse et explorations fonctionnelles rénales, Hopital Edouard Herriot, Hospices civils de Lyon, Lyon, France; INSERM 1060-CARMEN, Université de Lyon, Université Lyon 1, Lyon, France

**Keywords:** absorptive hypercalciuria, calcium load test, hypercalciuria

## Abstract

**Introduction:**

The calcium load test (CLT) was developed by Pak *et al.* in 1974 to better discriminate hypercalciuria. Absorptive hypercalciuria (AH) is defined by an increase of the difference between urinary calcium/creatinine ratio (ΔUCa/Cr) of more than 0.5 mmol/mmol with a 4-hour CLT. In clinical practice and more recent studies, CLT is a 2-hour test. We hypothesized that the 4 h timepoint is more efficient in AH diagnosis.

**Methods:**

We report a single-centre retrospective study including all patients who underwent CLT because of hypercalciuria or hyperparathyroidism. After a 3-day low-calcium diet and a 12-hour fast, 24-hour urines were collected. Blood and urinary samples were done at arrival and after 2 h and 4 h of oral ingestion of 1 g of calcium. AH was diagnosed by ΔUCa/Cr between baseline and 2 h or 4 h of more than 0.05 mmol/mmol.

**Results:**

We included 328 patients. Baseline UCa/Cr ratio was 0.3 ± 0.2 mmol/mmol and increased significantly after 2 h and 4 h (0.6 ± 0.3 and 0.8 ± 0.4 mmol/mmol, *P* < 0.001). ΔUCa/Cr was significantly different between baseline and 2 h or 4 h (0.2 ± 0.2 versus 0.5 ± 0.4, *P* < 0.001). AH was diagnosed in 35 (10.7%) patients after 2 h, 84 (25.6%) more were diagnosed at 4 h (*P* < 0.001).

**Conclusions:**

The 4 h CLT improves the diagnosis of AH with more than 50% of AH diagnosed within 4 h of calcium ingestion. It seems that there are cases of AH of later diagnosis with a similar clinical and biological profile depending on enteral absorption.

## Graphical Abstract

**Graphical Abstract:**
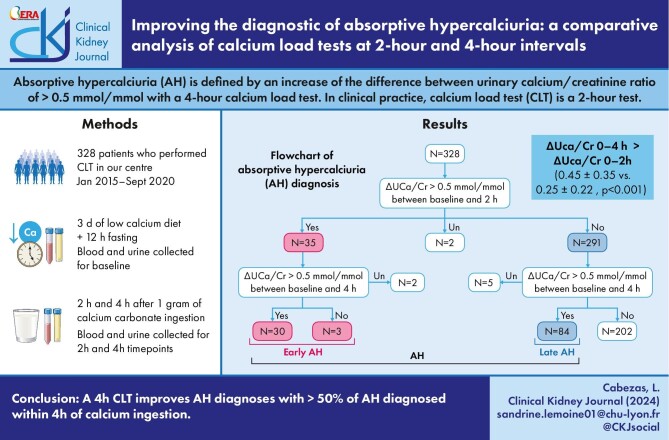

KEY LEARNING POINTS **What was known:**

- the calcium load test was developed by Pak *et al.* in 1974 to better discriminate hypercalciuria. Absorptive hypercalciuria is defined by an increase of the difference between urinary calcium/creatinine ratio of more than 0.5 mmol/mmol with a 4-hour calcium load test. In clinical practice and more recent studies, calcium load test is a 2-hour test.

**This study adds:**

- an improvement of absorptive hypercalciuria diagnosis at 4 hours of a calcium load test.

**Potential impact:**

- modification of calcium load test protocols for a 4-hour test and a better diagnosis of those patients.

## INTRODUCTION

The definition of absorptive, resorptive and renal hypercalciuria was described in the 1970s–1980s and was first reported by Pak *et al.* [1, 2]. Pak and Nicar corroborated this concept by demonstrating that patients with absorptive hypercalciuria (AH) or renal hypercalciuria exhibit distinct different biological profiles in terms of urinary calcium excretion, parathyroid hormone (PTH) levels, and 1,25-dihydroxyvitamin D [3]. As these diagnoses have led to tailored treatments for patients with kidney stones or primary hyperparathyroidism, a diagnostic test as calcium load test (CLT) was developed by Pak *et al.* in 1975 [4] to more accurately discriminate hypercalciuria.

This initial test was conducted on 109 patients (28 primary hyperparathyroidism, 24 patients with absorptive hypercalciuria, 6 with renal hypercalciuria, 24 with normocalciuric nephrolithiasis, and 27 controls). Despite subsequent descriptions of modifications to the CLT, such as variations in the low-calcium diet method [5] or different expressions of results [6], the original Pak test remains the established standard and has remained unchallenged. The test was conducted after seven low-calcium diet days (around 400 mg). Initially, the calcium load test involved a 2 h and 4 h evaluation of the difference in urinary calcium/creatinine ratio from baseline (ΔUCa/Cr). AH was defined by an increase in ΔUCa/Cr exceeding the 96th percentile range observed in healthy subjects after the fourth hour (i.e. surpassing 0.2 mg/mg or 0.5 mmol/mmol) [4]. Broadus *et al.* were the first to describe a 2 h evaluation because of the blood calcium level peak allowing a better hyperparathyroidism diagnosis without looking at AH diagnoses [6]. Even if in the first CLT described [4] there were no Ca/Cr differences in the AH population between 2 h and 4 h, it seems that the 4 h timepoint is essential for AH diagnosis defined by an increase of the calcium urinary excretion of more 0.20 mg calcium/100 ml of glomerular filtration between baseline and 4 h. In clinical practice and more recent studies, CLT is only a 2 h test using this 0.5 or 0.6 mmol/mmol cut-off as defined previously [7, 8] or an elevation of the UCa/Cr ratio of more than 0.158 mg/mg in paediatric cohorts [9, 10]. No other technical paper on the subject has been published since, and we still use the same protocol, although it has been performed with fewer than 30 patients in each group.

We hypothesized that a 2-h timepoint is not sufficient to diagnose all AH and that the 4-h timepoint, as initially described, is more efficient to improve accuracy of AH diagnosis. We aimed to establish that a 4 h CLT protocol is necessary when hyperabsorption is suspected.

## MATERIALS AND METHODS

### Patients

We report a single-centre retrospective study. All patients who underwent CLT between January 2015 and September 2020 in the Nephrology Department of the Lyon University Hospital were included, corresponding to two to three patients per week. No patients were excluded, even if the test was incomplete due to the lack of one dosing. These tests were carried out in patients with hypercalciuria (defined by a 24-h calcium urinary excretion of more than 0.10 mmol/kg/day or a concentration hypercalciuria of more than 3.8 mmol/l on a urinary sample) or in patients with elevated PTH (standard 15–65 ng/l, Elecsys PTH) with normocalcaemia, with or without a history of kidney stones. All patients included were over 18 years old and had performed an oral CLT in our centre. We excluded patients under 18 years old and patients for whom the CLT was not possible because of hypercalcaemia. Clinical [age, body mass index (BMI), sex] and biological data were collected retrospectively. Glomerular filtration rates (GFR) were estimated by the CKD-EPI formula.

All procedures were carried out in accordance with the ethical standards for single-centre retrospective studies. The study was conducted according to the guidelines of the Declarations of Helsinki and was approved by the relevant Institutional Review Boards (Hospital Edouard Herriot IRB# DC-2012–1615, dated 2 July 2012). All individuals gave informed consent before joining the study. This retrospective study was approved by the local Institutional Review Board (Comité d'Ethique des Hospices Civils de Lyon, MR004 no. 24–5156). The data that support the findings of this study are available from the corresponding author upon reasonable request.

### Calcium load test

After a low-calcium diet for 3 days and a 12-h fast, 24-h urines were collected for calcium, creatinine, urea, magnesium, and phosphate dosing. Blood and urinary samples were done at arrival for baseline values of calcaemia, phosphataemia, magnaesemia, PTH, and UCa/Cr levels. 25-Hydroxyvitamin D and 1,25-dihydroxyvitamin D3 were also assessed at this time. Resorptive hypercalciuria was diagnosed at this point if baseline UCa/Cr was higher than 0.35 mmol/mmol. Crystalluria was analysed within 2 h from the fresh urinary samples at arrival using polarization microscopy of urine homogenized by inversion. Two and 4 h after ingestion of 1 g of calcium carbonate per os, blood and urinary samples were collected for calcium, phosphate, PTH levels, and UCa/Cr evolution. AH was diagnosed by a ΔUCa/Cr at baseline and 2 h or 4 h timepoints of more than 0.5 mmol/mmol. The use and interpretation of the CLT are outlined in the Supplementary Fig. 1.

### Endpoints

Our primary endpoint was to compare the number of AH diagnosed after 2 h and 4 h of testing using a 0.5 mmol/mmol delta at each timepoint. UCa/Cr and ΔUCa/Cr evolution between baseline and 2 or 4 h was evaluated.

We also analysed clinical and biological profiles of early and late AH, defining the early AH by those diagnosed at 2 h timepoint and late AH as those diagnosed at the 4 h timepoint.

### Statistical analysis

Results are expressed as means with standard deviation and medians with the first and third quartile depending on the distribution. A Wilcoxon test was used for paired comparisons between two groups and Mann–Whitney for unpaired comparisons of two groups. Analysis of variance (ANOVA) or Kruskall Wallis tests were performed for the comparisons of more than 2 groups of patients. A two-sided *P*-value of <0.05 was considered statistically significant. Statistical analyses were conducted using the R statistical software 4.3.0 [11].

## RESULTS

### Population characteristics

A total of 328 patients were included. Our cohort was composed of 53% women, and the mean age was 48 ± 15 years. Mean GFR was 93 ± 22 ml/min/1.73 m^2^. Clinical and biological characteristics are described in Supplementary Table 1.

### Calcium load test results

CLT results are presented in Supplementary Table 2. At baseline, ionized calcium (iCa^2+^) levels were 1.24 ± 0.07 mmol/l with a significant increase at 2 h (1.28 ± 0.07 mmol/l, *P* < 0.001) and a stabilization at 4 h (1.28 ± 0.07 mmol/l, *P* = ns). Simultaneously, PTH showed a significant drop between baseline and 2 h (60 ± 26 ng/l versus 35 ± 19 ng/l, *P* < 0.001) followed by a significant increase between 2 and 4 h (35 ± 19 ng/l versus 39 ± 21 ng/l, *P* < 0.01). Mean 25-hydroxyvitamin D and 1,25-dihydroxyvitamin D3 were, respectively, 67 ± 24 nmol/l and 154 ± 54 pmol/l at baseline. Data distributions are presented in Supplementary Fig. 2.

**Figure 1:**
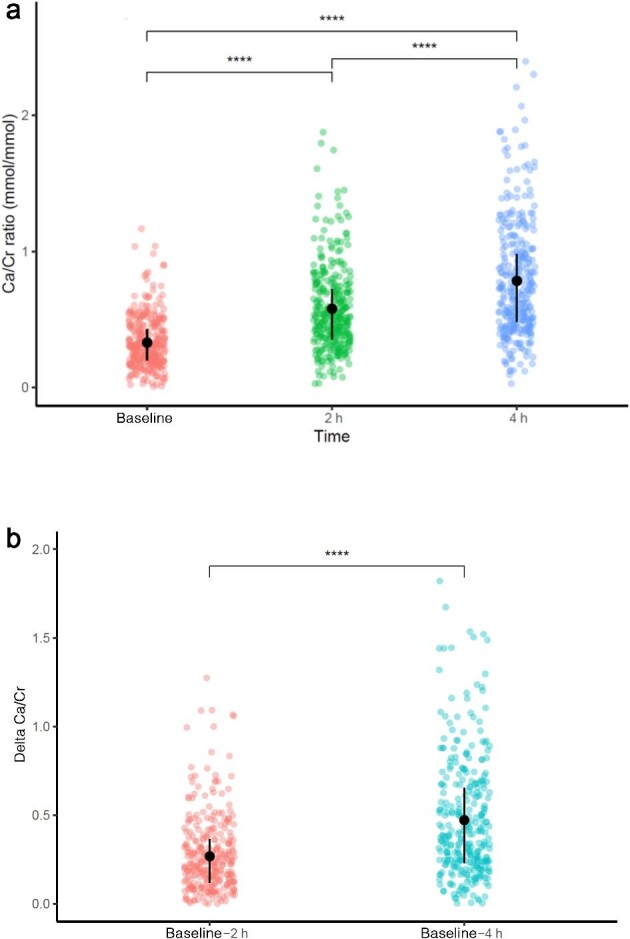
a. Urinary calcium/creatinine ratio at baseline and 2 and 4 h after CLT in the whole cohort. b. Difference of the urinary calcium/creatinine ratio between baseline and 2 h, and between baseline and 4 h in the whole cohort. A paired Wilcoxon test has been performed for paired comparison. ****=*P* < 0.00001.

Baseline UCa/Cr ratio was 0.33 ± 0.19 mmol/mmol and increased significantly after 2 h (0.58 ± 0.31 mmol/mmol, *P* < 0.001) and 4 h (0.78 ± 0.42 mmol/mmol, *P* < 0.001) (Fig. 1a). ΔUCa/Cr was significantly different between baseline and 2 h and between baseline and 4 h (0.25 ± 0.22 versus 0.45 ± 0.35, *P* < 0.001; Fig. 1b).

Phosphate levels showed a significant increase between baseline and 4 h (0.94 ± 0.18 mmol/l versus 1.02 ± 0.16 mmol/l, *P* < 0,0001). Phosphaturia/creatinine ratio showed a significant decrease between baseline and 4 h but no differences were found between baseline and 2 h and between, 2 h and 4 h (1.54 ± 0.69 mmol/mmol versus 1.41 ± 0.64 mmol/mmol between baseline and 4 h, *P* < 0.05; 1.54 ± 0.69 versus 1.49 ± 0.68 mmol/l between 2 h and 4 h, *P* = ns). Other data distributions are presented in Supplementary Fig. 2.

### Difference of AH diagnosis between 2 h and 4 h after CLT

**Figure 2:**
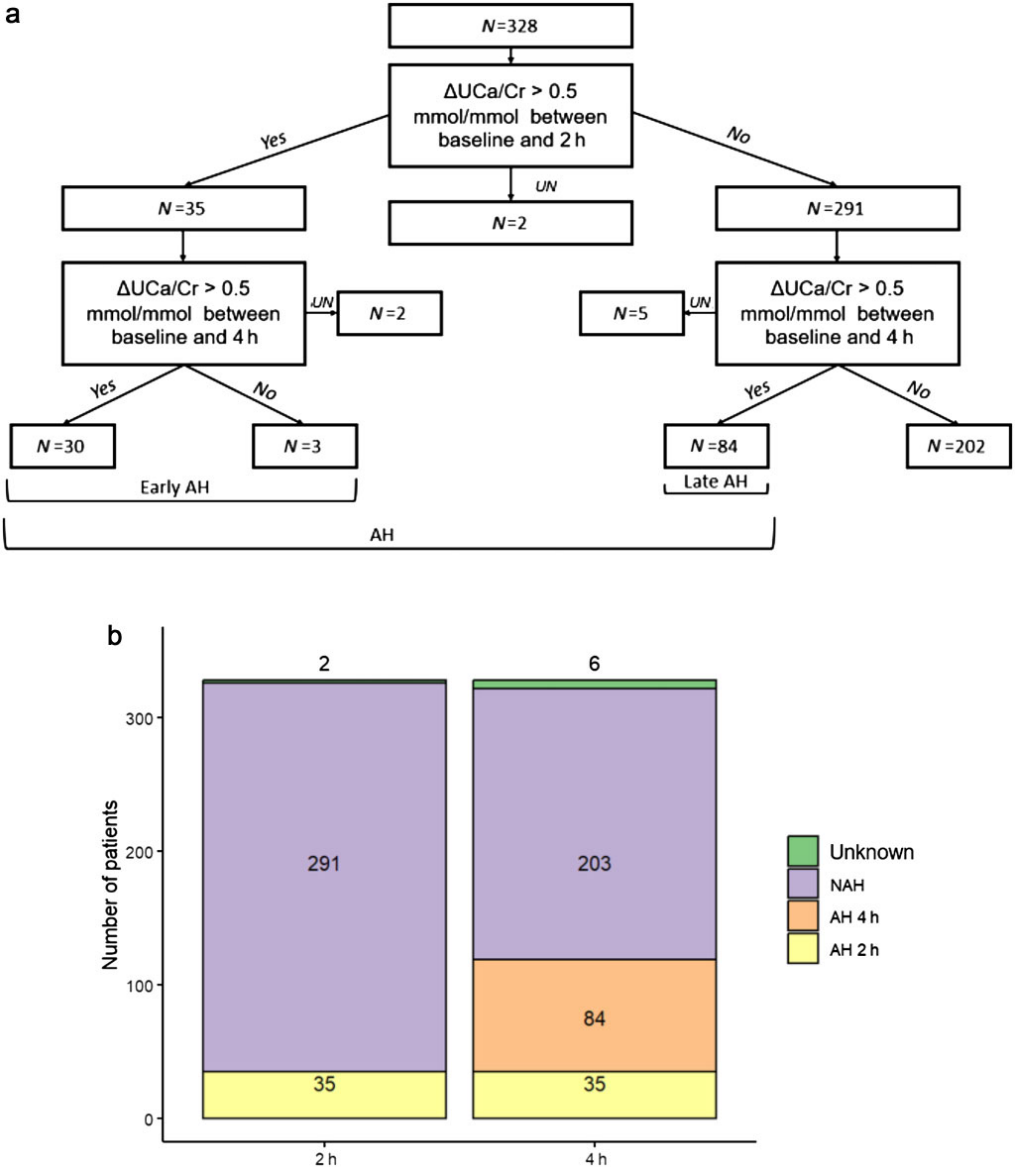
a. Flowchart of the absorptive hypercalciuria (AH) diagnosis. AH = absorptive hypercalciuria. UN = unknown. Early AH represents patients that were diagnosed after 2 h of the calcium load test and late AH represents patients that were diagnosed after 4 h. b. AH diagnosis at 2 h and 4 h. A chi-sqaured test was performed between AH 2 h and AH 4 h groups with a *P*-value < 0.0001. UN = unknown, NAH = no absorptive hypercalciuria, AH 2 h = absorptive hypercalciuria at 2 h, and AH 4 h = absorptive hypercalciuria at 4 h.

AH was diagnosed in 35 (10.7%) patients based on an increase of ΔUCa/Cr > 0.5 mmol/mmol between baseline and 2 h. An additional 84 patients were diagnosed based on an increase of ΔUCa/Cr > 0.5 mmol/mmol between baseline and 4 h. A total of 119 (36.3%) AH were diagnosed when we included analysis of UCa/Cr at 2 h and 4 h (Fig. 2a and Fig. 2b).

Only three patients had a ΔUCa/Cr > 0.5 mmol/mmol between baseline and 2 h but which wasn't elevated between baseline and 4 h (Fig. 2).

Based on the Ca/Cr ratio at baseline, after 12-h fasting and a low-calcium diet for 3 days, 97 patients (30%) were diagnosed with resorptive hypercalciuria (baseline UCa/Cr higher than 0.35 mmol/mmol).

#### Clinical and biological patient profiles between AH and no AH

In comparison to the rest of the cohort, AH were women for 68.1% [versus 43.6% in the no absorptive hypercalciuria (NAH) group, *P* < 0.001]. There was no age or BMI difference between the two groups. GFR was significantly higher in the AH group [102.0 (91.3, 112.2) versus 90.8 (70.1, 105.5) ml/min/1.73 m^2^, *P* < 0.001].

**Figure 3:**
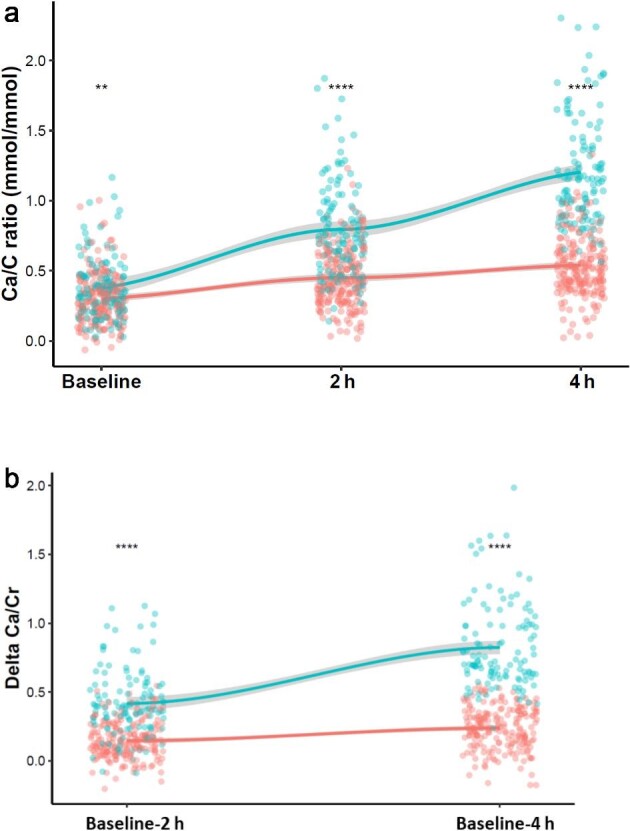
Changes of the a. Ca/Cr ratio and b. delta Ca/Cr across the CLT between baseline, 2 h and 4 h and in the AH (blue) and no AH (red) group. A paired Wilcoxon test has been performed for paired comparison. ns = not significant, *=*P* < 0.01, **=*P* < 0.001, ***=*P* < 0.0001 ****=*P* < 0.00001.

As expected, AH had a higher 1,25-dihydroxyvitamin D3 level [167.5 (127.5, 201.2) versus 140.0 (109.8, 174.0) pg/ml, *P* < 0.001]. The AH group displayed a higher calcaemia at 2 h and 4 h associated with a lower PTH at 2 h and 4 h and lower phosphate level at 2 h. Results are presented in Table 1 and data distribution across CLT are presented in Fig. 3 and Supplementary Fig. 3.

**Table 1:** Clinical and biological profile of AH and no AH patients.

	NAH (*N* = 202)	AH (*N* = 119)	*P*-value
**Clinical**			
Gender, *N* (%)			**<0.001**
Female	88 (43.6%)	81 (68.1%)	
Male	114 (56.4%)	38 (31.9%)	
Age (year), median (Q1, Q3)	47.86 (13.95)	48.79 (15.79)	0.146
BMI (kg/m²), mean (SD)	21.99 (1.47)	22.05 (1.46)	0.074
**Serum analyses**	**Mean (SD)**		***P*-value**
Creatininaemia (µmol/l)	77.9 (19.9)	64.9 (11.4)	**<0.001**
Glomerular filtration rate (ml/min/1.73m²)	88 (23)	101 (18)	**<0.001**
HCO_3_^–^ (mmol/l)	25.1 (2.4)	24.8 (2.1)	0.557
Uric acid (mmol/l)	318 (79)	287 (71)	**0.003**
25-Hydroxyvitamin D (nmol/l)	69.0 (26.9)	65.0 (20.5)	0.452
1,25-Dihydroxyvitamin D3 (pmol/l)	144.1 (47.3)	170.7 (57.4)	**<0.001**
Magnesaemia (mmol/l)	0.82 (0.07)	0.81 (0.07)	0.555
Total calcium (mmol/l) 0 h	2.37 (0.13)	2.38 (0.13)	0.645
Total calcium (mmol/l) 2 h	2.42 (0.14)	2.50 (0.14)	**<0.001**
Total calcium (mmol/l) 4 h	2.44 (0.14)	2.51 (0.15)	**<0.001**
Ionized calcium (mmol/l) 0 h	1.23 (0.07)	1.24 (0.07)	0.621
Ionized calcium (mmol/l) 2 h	1.26 (0.06)	1.30 (0.07)	**<0.001**
Ionized calcium (mmol/l) 4 h	1.26 (0.06)	1.31 (0.06)	**<0.001**
Parathyroid hormone (ng/l) 0 h##	60.3 (26.2)	60.0 (25.9)	0.471
Parathyroid hormone (ng/l) 2 h	38.4 (20.3)	30.1 (17.3)	**<0.001**
Parathyroid hormone (ng/l) 4 h	41.4 (20.7)	36.5 (22.3)	**0.019**
Phosphate (mmol/l) 0 h	0.93 (0.17)	0.94 (0.18)	0.619
Phosphate (mmol/l) 2 h	0.88 (0.16)	0.90 (0.16)	**0.006**
Phosphate (mmol/l) 4 h	1.00 (0.16)	1.05 (0.16)	**0.014**
**Urine analyses**	**Mean (SD)**		***P*-value**
24 h urine volume (ml/day)	1888 (728)	1996 (697)	0.339
Urinary density (first morning spot urine)	1027 (0.01)	1024 (0.01)	0.045
Urinary pH (first morning spot urine)	5.85 (0.71)	5.87 (0.72)	0.400
Ca/Cr (mmol/mmol) 0 h	0.30 (0.17)	0.38 (0.21)	0.006
Ca/Cr (mmol/mmol) 2 h	0.45 (0.20)	0.80 (0.34)	**<0.001**
Ca/Cr (mmol/mmol) 4 h	0.54 (0.20)	1.20 (0.37)	**<0.001**
ΔCa/Cr 0–2 h	0.15 (0.13)	0.42 (0.23)	**<0.001**
ΔCa/Cr 0–4 h	0.24 (0.14)	0.82 (0.29)	**<0.001**
Tmp/GFR	0.81 (0.17)	0.84 (0.18)	0.185

Bold entries are correspond to significant results.

Urinary pH and urinary density have been measured on urinary samples at arrival, right before the CLT.

#### Clinical and biological patients’ profile between 2 h AH and 4 h AH

**Figure 4:**
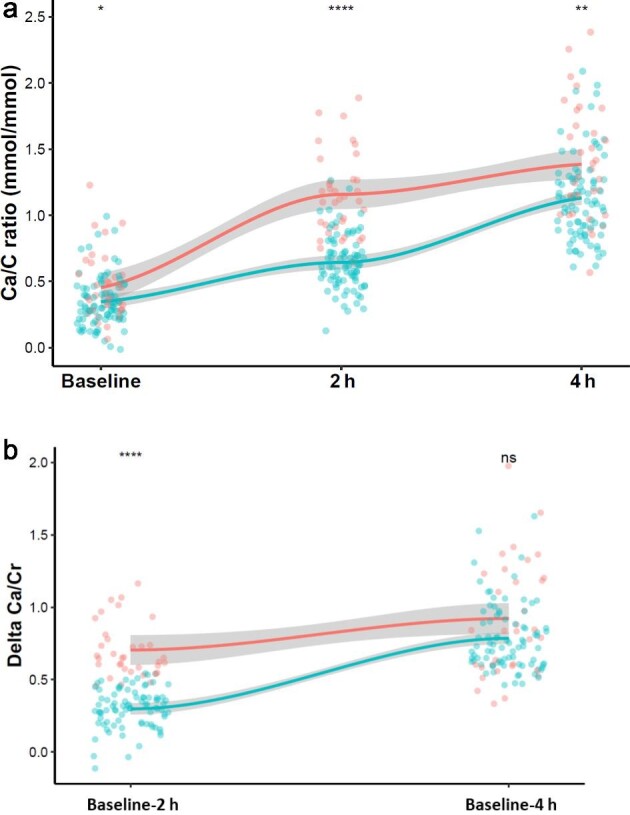
Changes of the a. Ca/Cr ratio and b. delta Ca/Cr across the CLT between baseline, 2 h and 4 h and in the early AH (red) and late AH (blue) group. A paired Wilcoxon test has been performed for paired comparison. ns = not significant, *=*P* < 0.01, **=*P* < 0.001, ***=*P* < 0.0001 ****=*P* < 0.00001.

Between early and late AH, we found no significant differences in 1,25-dihydroxyvitamin D3, circulating and urinary phosphate, but 25-hydroxyvitamin D was higher in late AH (67.3 ± 21 ng/ml in late AH versus 59.2 ± 18.3 ng/ml, *P* = 0.039). Total and ionized calcium levels were higher in the early AH group at baseline, 2 h and 4 h for total calcium. No other differences were observed in clinical and biological data such as BMI, gender, age, or kidney function. Results are presented in Table 2 and data distribution across CLT are presented in Fig. 4 and Supplementary Fig. 4.

**Table 2:** Clinical and biological profile of early AH and late AH patients.

	Early AH (*N* = 35)	Late AH (*N* = 84)	*P*-value
**Clinical**			
Gender, *N* (%)			0.939
Female	24 (68.6%)	57 (67.9%)	
Male	11 (31.4%)	27 (32.1%)	
Age (year), median (Q1, Q3)	50.1 (14.8)	48.3 (16.2)	0.690
BMI (kg/m²), mean (SD)	22.20 (1.43)	21.99 (1.47)	0.590
**Serum analyses**	**Mean (SD)**		***P*-value**
Creatininaemia (µmol/l)	64 (11)	65 (12)	0.676
GFR (ml/min/1,73m²)	100 (17)	101 (18)	0.775
HCO_3_^–^ (mmol/l)	24.8 (2.2)	24.8 (2.1)	0.925
Uric acid (mmol/l)	289 (78)	286 (69)	0.976
25-Hydroxyvitamin D (nmol/l)	59.2 (18.3)	67.4 (21.0)	0.039
1,25-Dihydroxyvitamin D3 (pmol/l)	186.1 (71.9)	164.1 (48.9)	0.083
Magnesaemia (mmol/l)	0.82 (0.07)	0.81 (0.07)	0.797
Total calcium (mmol/l) 0 h	2.42 (0.13)	2.36 (0.13)	**0.021**
Total calcium (mmol/l) 2 h	2.56 (0.15)	2.48 (0.13)	**0.016**
Total calcium (mmol/l) 4 h	2.56 (0.16)	2.50 (0.15)	**0.040**
Ionized calcium (mmol/l) 0 h	1.28 (0.08)	1.23 (0.05)	**0.010**
Ionized calcium (mmol/l) 2 h	1.34 (0.08)	1.29 (0.05)	**<0.001**
Ionized calcium (mmol/l) 4 h	1.33 (0.08)	1.30 (0.06)	0.075
Parathyroid hormone (ng/l) 0 h	62.2 (31.6)	59.1 (23.2)	0.838
Parathyroid hormone (ng/l) 2 h	33.7 (23.2)	28.7 (14.1)	0.544
Parathyroid hormone (ng/l) 4 h	44.8 (30.0)	33.3 (17.7)	0.078
Phosphate (mmol/l) 0 h	0.96 (0.21)	0.93 (0.16)	0.924
Phosphate (mmol/l) 2 h	0.92 (0.15)	0.89 (0.16)	0.357
Phosphate (mmol/l) 4 h	1.06 (0.14)	1.04 (0.17)	0.697
**Urine analyses**	**Mean (SD)**		***P*-value**
24 h urine volume (ml/day)	2130 (690)	1940 (697)	0.135
Urinary density (first morning spot urine)	1024 (0.01)	1023 (0.01)	0.594
Urinary pH (first morning spot urine)	5.86 (0.78)	5.88 (0.69)	0.702
Ca/Cr (mmol/mmol) 0 h	0.45 (0.24)	0.35 (0.19)	**0.013**
Ca/Cr (mmol/mmol) 2 h	1.16 (0.30)	0.64 (0.21)	**<0.001**
Ca/Cr (mmol/mmol) 4 h	1.39 (0.43)	1.13 (0.31)	**0.003**
ΔCa/Cr 0–2 h	0.70 (0.19)	0.30 (0.12)	**<0.001**
ΔCa/Cr 0–4 h	0.92 (0.39)	0.78 (0.23)	0.121
Tmp/GFR	0.84 (0.21)	0.84 (0.16)	0.882

Bold entries are correspond to significant results.

Urinary pH and urinary density have been measured on urinary samples at arrival, right before the CLT.

## DISCUSSION

We present here the first study comparing AH diagnoses at 2 h and 4 h of a CLT. We found that two-thirds of AH were diagnosed at 4 h whereas this CLT is performed in most studies and laboratories with only a 2-h timepoint. There is a lack of data in the literature about CLT and the main references remain the initial Pak test described in 1975 [4].

Initially, in PAK's study [4], AH diagnosis was established through the recovery of ^47^Ca in the faeces following oral ingestion of the isotope. CLT was conducted in the same cohort of patients, revealing a mean increase of 0.2 mg/mg (0.5 mmol/mmol) of calcium to creatinine ratio in a 4-h urine collection. Based on these findings, a proposed urinary Ca/Cr ratio of 0.5 mmol/mmol (0.2 mg/mg) was established. Despite its widespread use, the cut-off value for Ca/Cr of 0.5 mmol/mmol (0.2 mg/mg) has been maintained for both 2-h and 4-h collections. Peacock *et al*. showed also a peak for serum calcium and urinary calcium between 3 and 4 h after CLT, higher for stone formers [12]. In this study, we confirmed that reducing the urine collection for a 2 h urine Ca/Cr ratio results in a decrease in the number of AH diagnoses.

Using the same Pak criteria to diagnose AH (AH diagnosed if mean ΔUCa/Cr between baseline – 2 h and baseline – 4 h is higher than 0.5 mmol/mmol), we were able to diagnose more AH patients than with the ΔUCa/Cr between baseline and 2 h alone. This supports the idea that a 4 h timepoint is essential for AH diagnosis. In addition, because three patients were diagnosed with AH at 2 h but not at 4 h, we suggest that the 2 h timepoint does have its importance, probably even more for the hyperparathyroidism diagnoses. Indeed, plasmatic calcium was the higher at 2 h and PTH the lower at 2 h.

These results may be explained by variations in calcium absorption in the digestive system, which can differ among patients. Intestinal calcium absorption occurs through a transcellular way and a paracellular pathway, both regulated by hormones, nutrients, and other factors, such as PTH, Klotho, and mainly dihydroxyvitamin D through TRPV6 [13]. We hypothesized that calcium absorption can vary between patient link intestinal pH [14]. This factor can be linked to associated treatment like proton pump inhibitors, diet, and comorbidities. One of the first intestinal calcium absorption descriptions was in 1969 by Birge *et al*., whom developed a simple test using calcium isotope stool measure to estimate intestinal calcium absorption [15]. They first described that after 2.5 h, 95% of calcium absorption was complete. Another test was described in 1987 by Milsom *et al*. using the measure of stable strontium after 4 h ingestion in order to estimate intestinal calcium absorption [16]. This test was used in a cohort of 172 hypercalciuric stone formers, 36 hypercalciuric non-stone formers, and 40 normocalciuric controls by Vezzoli *et al*. [17]. They showed, using a 4 h test, that serum strontium concentration was higher in the hypercalciuric stone-former group and that strontium clearance was also greater in hypercalciuric stone-former patients. No study comparing 2 h and 4 h calcium intestinal absorption has been done recently, but all tests described were performed after 4 h ingestion. A 4 h test would permit to increase our diagnostic capabilities.

In a more recent study by Tostivint *et al*., AH was diagnosed in the presence of ΔUCa/Cr > 0.60 mmol/mmol after 2 h CLT [8]. Concerning the 90 min urinary measurements, the ΔUCa/Cr was significantly higher in the AH group as compared to the others. Our results differ, first because we performed a longer test; and second, looking at ionized calcium changes between baseline, 2 h and 4 h and comparing the early AH and late AH group, we can see that in the early AH group, baseline ionized calcium was significantly higher than in the late AH group. We also can observe that at 4 h, ionized calcium is stable in the early AH group but is still increasing in the late AH group. This supports the idea of two types of intestinal calcium absorbers, slow ones and fast ones. In our cohort, using the 2 h and 4 h test we diagnosed 119 AH over 328 patients corresponding to 36% of the cohort, which is similar to the literature. Tostivint *et al*. found 40% of AH in their cohort [8] and 33% of AH above hypercalciuric children in the Kruse *et al*. cohort [9].

Other methods can be used to improve AH diagnosis. First, dietary interventions about salt or calcium ingestion can be done, evaluated by CLT before and after, in order to eliminate dietary hypercalciuria. Some genetic studies can be performed, especially when there is family history of stones or hypercalciuria, vitamin D receptor and calcium sensor receptor genes [18]. CLT can help phenotyping our patients, leading to a more precise diagnosis.

Our study presents some limits. It is a monocentric study using a retrospective cohort. Some may be linked to the CLT and to the low-calcium diet that we were unable to be sure of. Patients needed to be on a low-calcium diet, less than 400 mg, 3 days before the CLT. The low-calcium diet was explained by the doctor on the day of the CLT programming and a list of foods to avoid was given to the patients. All patients had non-dietetic hypercalciuria diagnosed before the CLT. Other methods have been described, such as the use of sodium cellulose phosphate to chelate calcium in the intestine and avoid calcium absorption [5]. This was described once and is not used in our centre. Another limitation of this study is the cut-off definition. These cut-offs are based on studies dating from the 1980s and have been used since then, but in a different way for each team. In this study, we were using the same cut-off at 2 h and 4 h for AH diagnoses. Looking at intestinal calcium absorption, cut-offs may be lower at 2 h than at 4 h [19], but other isotopic tests are needed to compare 2 h and 4 h intestinal absorption. Finally, resorptive hypercalciuria (including hyperparathyroidism) and renal hypercalciuria can also be associated with AH due to increased calcitriol levels. Unfortunately, because of the design of our study, we were not able to have the patients’ final diagnoses. This point doesn't change the fact that AH, with or without renal or resorptive hypercalciuria, is better diagnosed after a 4h-CLT and can lead to specific recommendations.

## TAKE HOME MESSAGES

This study shows that performing the 4 h CLT improves the diagnosis of AH with more than 50% of AH diagnosed within 4 h of calcium ingestion. It seems that there are cases of AH of later diagnosis with a similar clinical and biological profile depending on enteral absorption.

## Supplementary Material

sfae399_Supplemental_File

## Data Availability

The data underlying this article are available in the article and in its online supplementary material.
