# Supplementary material for: Improving the diagnostic of absorptive hypercalciuria: a comparative analysis of calcium load tests at 2-hour and 4-hour intervals
Source: Clin Kidney J. 2024 Dec 9;18(2):sfae399. doi: 10.1093/ckj/sfae399 (PMC11799772; doi:10.1093/ckj/sfae399)
Supplement: sfae399_Supplemental_File [file sfae399_supplemental_file.pdf]

SUPPLEMENTARY DATA

STROBE Statement—checklist of items that should be included in reports of observational studies

	Item No	Recommendation
**Title and abstract**	1	(*a*) Indicate the study's design with a commonly used term in the title or the abstract
		(*b*) Provide in the abstract an informative and balanced summary of what was done and what was found
**Introduction**		
Background/rationale	2	Explain the scientific background and rationale for the investigation being reported
Objectives	3	State specific objectives, including any prespecified hypotheses
**Methods**		
Study design	4	Present key elements of study design early in the paper
Setting	5	Describe the setting, locations, and relevant dates, including periods of recruitment, exposure, follow-up, and data collection
Participants	6	(*a*) *Cohort study*—Give the eligibility criteria, and the sources and methods of selection of participants. Describe methods of follow-up<br>*Case-control study*—Give the eligibility criteria, and the sources and methods of case ascertainment and control selection. Give the rationale for the choice of cases and controls<br>*Cross-sectional study*—Give the eligibility criteria, and the sources and methods of selection of participants
		(*b*) *Cohort study*—For matched studies, give matching criteria and number of exposed and unexposed<br>*Case-control study*—For matched studies, give matching criteria and the number of controls per case
Variables	7	Clearly define all outcomes, exposures, predictors, potential confounders, and effect modifiers. Give diagnostic criteria, if applicable
Data sources/ measurement	8*	For each variable of interest, give sources of data and details of methods of assessment (measurement). Describe comparability of assessment methods if there is more than one group
Bias	9	Describe any efforts to address potential sources of bias
Study size	10	Explain how the study size was arrived at
Quantitative variables	11	Explain how quantitative variables were handled in the analyses. If applicable, describe which groupings were chosen and why
Statistical methods	12	(*a*) Describe all statistical methods, including those used to control for confounding
		(*b*) Describe any methods used to examine subgroups and interactions
		(*c*) Explain how missing data were addressed
		(*d*) *Cohort study*—If applicable, explain how loss to follow-up was addressed<br>*Case-control study*—If applicable, explain how matching of cases and controls was addressed<br>*Cross-sectional study*—If applicable, describe analytical methods taking account of sampling strategy
		(*e*) Describe any sensitivity analyses

Continued on next page

**Results**		
Participants	13*	(a) Report numbers of individuals at each stage of study—eg numbers potentially eligible, examined for eligibility, confirmed eligible, included in the study, completing follow-up, and analysed
		(b) Give reasons for non-participation at each stage
		(c) Consider use of a flow diagram
Descriptive data	14*	(a) Give characteristics of study participants (eg demographic, clinical, social) and information on exposures and potential confounders
		(b) Indicate number of participants with missing data for each variable of interest
		(c) *Cohort study*—Summarise follow-up time (eg, average and total amount)
Outcome data	15*	*Cohort study*—Report numbers of outcome events or summary measures over time
		Case-control study—Report numbers in each exposure category, or summary measures of exposure
		Cross-sectional study—Report numbers of outcome events or summary measures
Main results	16	(*a*) Give unadjusted estimates and, if applicable, confounder-adjusted estimates and their precision (eg, 95% confidence interval). Make clear which confounders were adjusted for and why they were included
		(*b*) Report category boundaries when continuous variables were categorized
		(*c*) If relevant, consider translating estimates of relative risk into absolute risk for a meaningful time period
Other analyses	17	Report other analyses done—eg analyses of subgroups and interactions, and sensitivity analyses
**Discussion**		
Key results	18	Summarise key results with reference to study objectives
Limitations	19	Discuss limitations of the study, taking into account sources of potential bias or imprecision. Discuss both direction and magnitude of any potential bias
Interpretation	20	Give a cautious overall interpretation of results considering objectives, limitations, multiplicity of analyses, results from similar studies, and other relevant evidence
Generalisability	21	Discuss the generalisability (external validity) of the study results
**Other information**		
Funding	22	Give the source of funding and the role of the funders for the present study and, if applicable, for the original study on which the present article is based

*Give information separately for cases and controls in case-control studies and, if applicable, for exposed and unexposed groups in cohort and cross-sectional studies.

Note: An Explanation and Elaboration article discusses each checklist item and gives methodological background and published examples of transparent reporting. The STROBE checklist is best used in conjunction with this article (freely available on the Web sites of PLoS Medicine at http://www.plosmedicine.org/, Annals of Internal Medicine at http://www.annals.org/, and Epidemiology at http://www.epidem.com/). Information on the STROBE Initiative is available at www.strobe-statement.org.

4-hours calcium load test for absorptive hypercalciuria

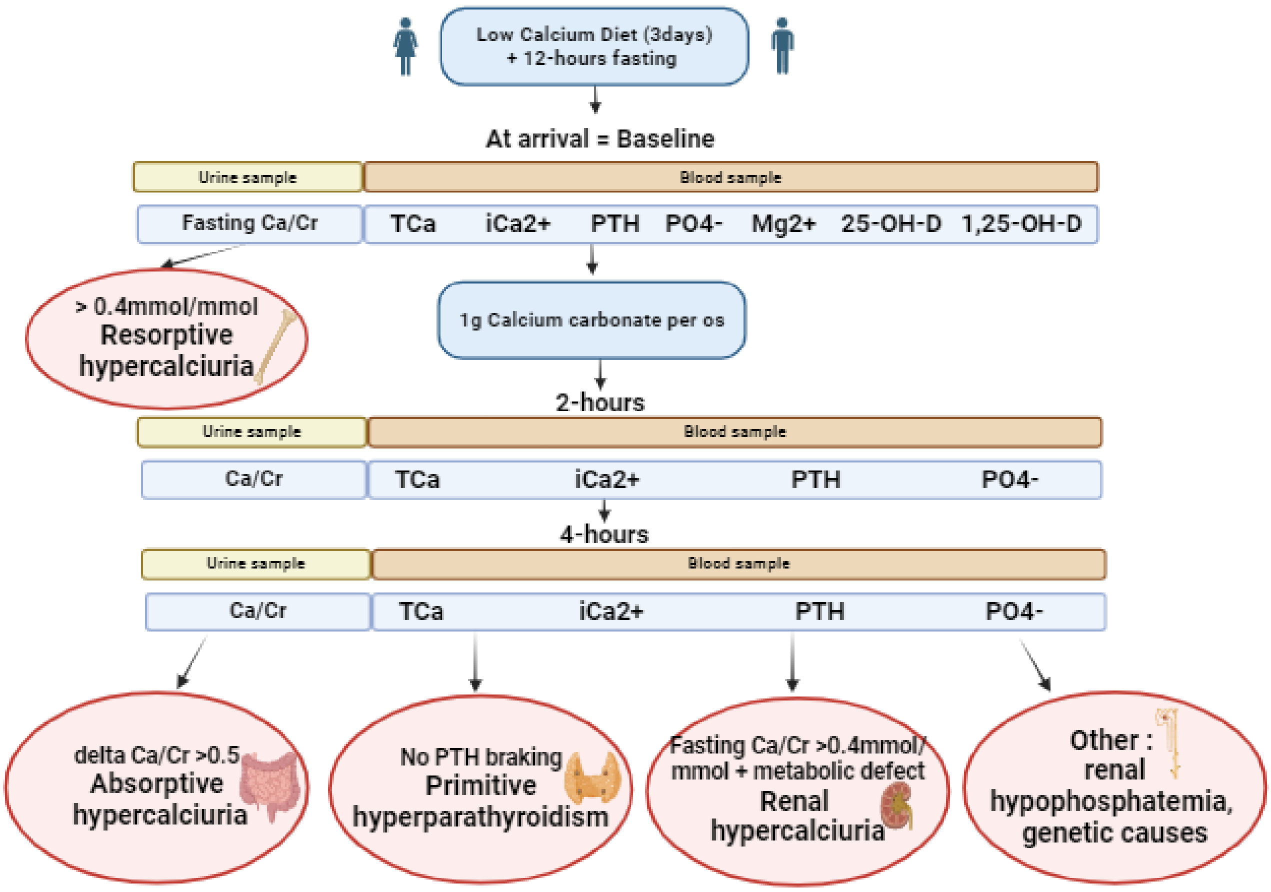

Supplementary Figure 1: Schematic representation of the use and interpretation of a calcium load test (CLT). Patients arrived at the hospital after 3 days of low calcium diet and 12-hours fasting. At arrival, urinary and blood samples were collected. After 3 days of low calcium diet, the first urinary calcium/creatinine (Ca/Cr) ratio reflect the bone resorption. If the Ca/Cr ratio is higher than 0.35 mmol/mmol, resorptive hypercalciuria is diagnosed. One gram calcium carbonate is given to the patient and after 2-hours and 4-hours, blood and urinary samples are collected. Absorptive hypercalciuria is diagnosed if the difference between Ca/Cr ratio at baseline and 2h or 4h is higher than 0.5. Primitive hyperparathyroidism is diagnosed if the parathormone (PTH) is not slowed even if the ionized calcium serum (iCa2+) is increased by the load (of more than 0.03mmol/L). Renal hypercalciuria is diagnosed if the fasting Ca/Cr ratio is higher than 0.35 mmol/mmol and if there are metabolic defects such as acidosis, hypomagnesemia, hypophosphatemia. This is a non-exhaustive list and others

diagnoses can be suspected with this CLT like renal hypophosphatemia or others calcium regulation diseases. TCa = Total calcium serum, PO4- = phosphatemia, Mg2+ = magnesemia, 25-OH-vitD = 25-Hydroxyvitamin D, 1,25-OH-vitD = 1,25-Dihydroxyvitamin D.

Supplementary Table 1: Demographic and Clinical Characteristics of the Participants at Baseline

	Total (N=328)
Clinical	
Gender, N (%)	
Female	173 (52.7%)
Male	155 (47.3%)
Age (year), Mean (SD)	48 (15)
BMI (kg/m²), Mean (SD)	22 (2)
Biology	
**Blood samples**	
Creatininemia (µmol/L), Mean (SD)	73 (18)
Estimated glomerural filtration rate (mL/min/1,73m²), Mean (SD)	93 (22)
Urea (mmol/L), Mean (SD)	5.0 (1.5)
HCO_3^- (mmol/L), Mean (SD)	25 (2.31)
Uric acid (mmol/L), Mean (SD)	306 (78)
**First morning spot urine**	
Urinary density, Mean (SD)	1020 (10)
Urinary pH, Mean (SD)	5.9 (0.7)
Cristalluria, N (%)	
Weddelite	55 (16,8)
Whewellite + Weddelite	8 (2,4)
Weddelite + Urate	1 (0,3)
Weddelite + Brushite	1 (0,3)
Brushite	1 (0,3)
Brushite + Amorphous Phosphate	1 (0,3)
Urate	3 (0,9)
Amorphous Phosphate	11 (3,4)
**24hrs urine**	
24hrs urine volume (mL/day), Mean (SD)	1926 (715)
Albuminuria (mg/L), Mean (SD)	45 (143)
Proteinuria (g/L), Mean (SD)	7.5 (15.7)

Cristalluria, Urinary pH and urinary density have been measured on urinary samples at arrival, right before the CLT. HCO_3- = bicarbonatemia, SD= standard deviation

Supplementary Table 2: Calcium load test results

Parameter		Total (N=328)	
**Serum analyses**	**Standards**	**Mean (SD)**	**p-value***
25-Hydroxyvitamin D (nmol/L)	>50	67.4 (24.7)	-
1,25-Dihydroxyvitamin D3 (pmol/L)	69-200	154.6 (54.1)	-
Magnesemia (mmol/L)	0.66-1.07	0.82 (0.07)	-
Total calcium (mmol/L) 0h	2.10-2.55	2.37 (0.13)	-
Total calcium (mmol/L) 2h		2.45 (0.15)	p<0.001
Total calcium (mmol/L) 4h		2.47 (0.15)	p<0.001
Ionized calcium (mmol/L) 0h	1.18-1.32	1.24 (0.07)	-
Ionized calcium (mmol/L) 2h		1.28 (0.07)	p<0.001
Ionized calcium (mmol/L) 4h		1.28 (0.07)	p<0.001
Parathormone (ng/L) 0h	15-65	60.0 (26.1)	-
Parathormone (ng/L) 2h		35.3 (19.7)	p<0.001
Parathormone (ng/L) 4h		39.5 (21.4)	p<0.001
Phosphate (mmol/L) 0h	0.74-1.52	0.94 (0.18)	-
Phosphate (mmol/L) 2h		0.89 (0.16)	p<0.01
Phosphate (mmol/L) 4h		1.02 (0.16)	p<0.001
**Urine analyses**			
Ca/Cr (mmol/mmol) 0h	<0,35	0.33 (0.19)	-
Ca/Cr (mmol/mmol) 2h	-	0.58 (0.31)	p<0.001
Ca/Cr (mmol/mmol) 4h	-	0.78 (0.42)	p<0.001
ΔCa/Cr 0-2h	<0.50	0.25 (0.22)	-
ΔCa/Cr 0-4h		0.45 (0.35)	p<0.001
Tmp/GFR	0.8-1.35	0.82 (0.17)	-

*Kruskall-wallis test has been performed between baseline and 2h or between baseline and 4h except for delta Ca/Cr, Kruskall-wallis test has been performed between the 0-2h delta and the 0-4h delta.

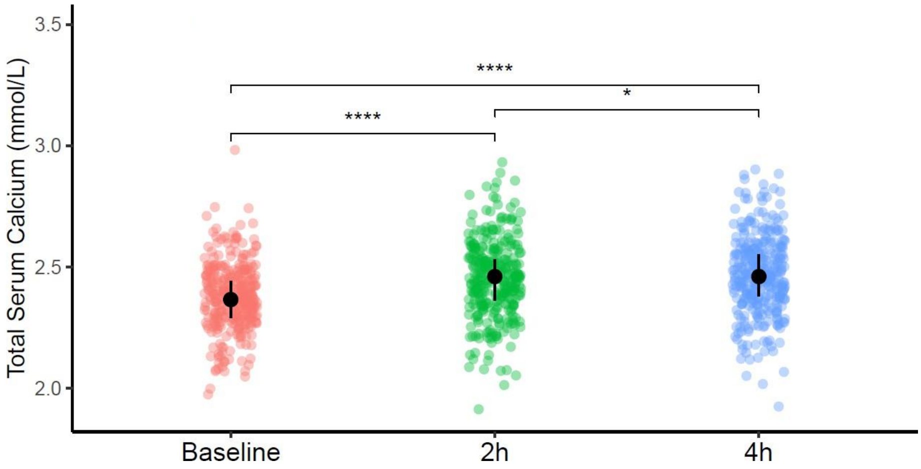
Total Serum Calcium (mmol/L)
3.5
3.0
2.5
2.0
****
*
****
Baseline
2h
4h

A.

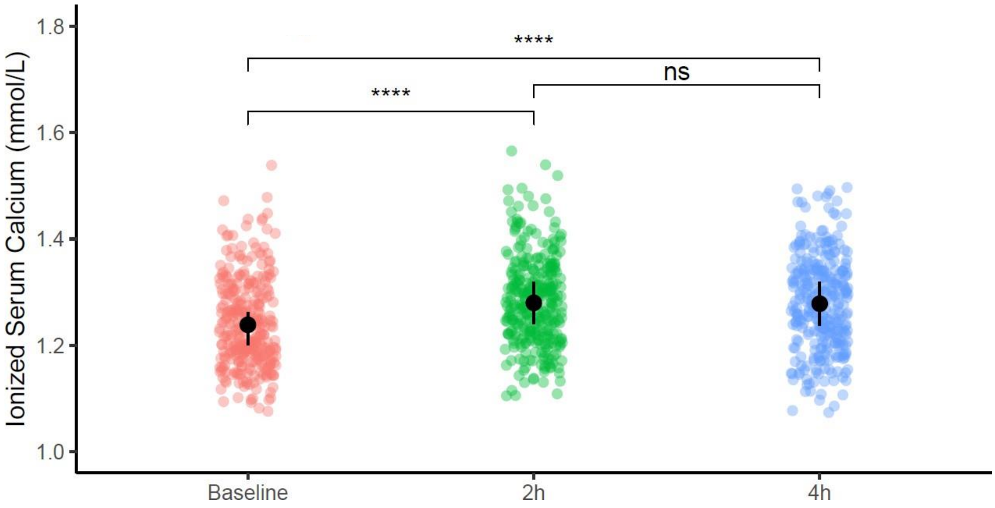
Ionized Serum Calcium (mmol/L)
1.8
1.6
1.4
1.2
1.0
****
ns
****
Baseline
2h
4h

B.

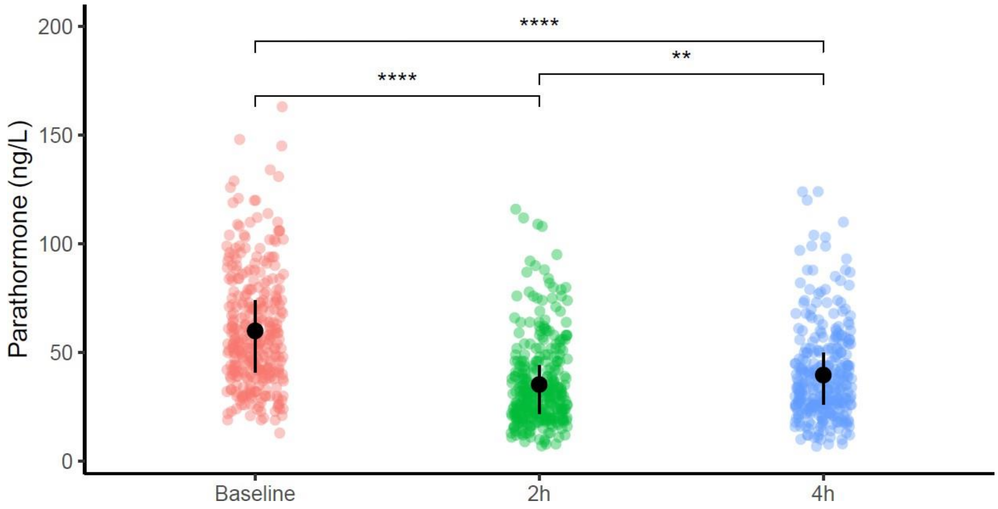
Parathormone (ng/L)
200
150
100
50
0
Baseline
2h
4h
****
**
****

C.

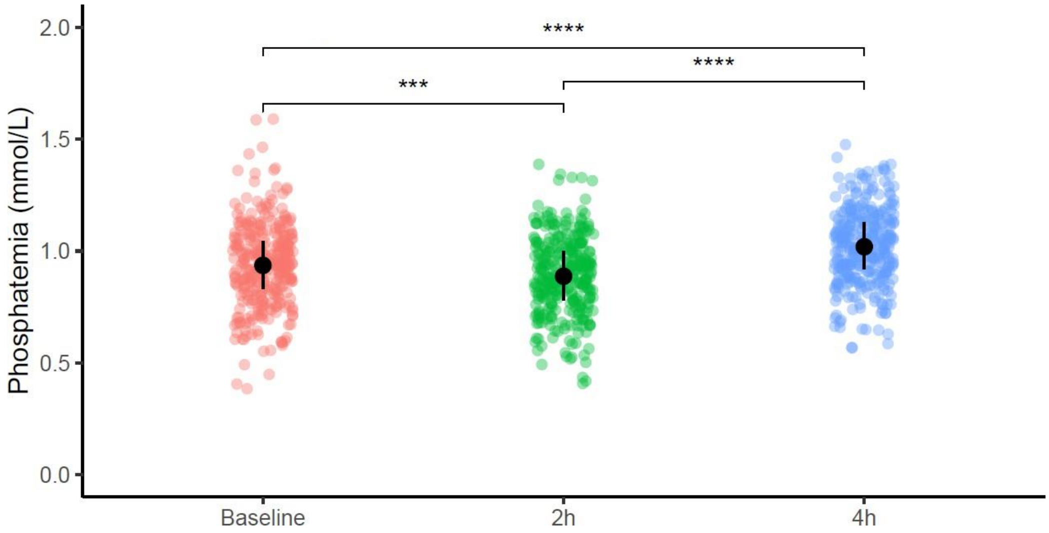
Phosphatemia (mmol/L)
2.0
1.5
1.0
0.5
0.0
Baseline
2h
4h
****
****
***

D.

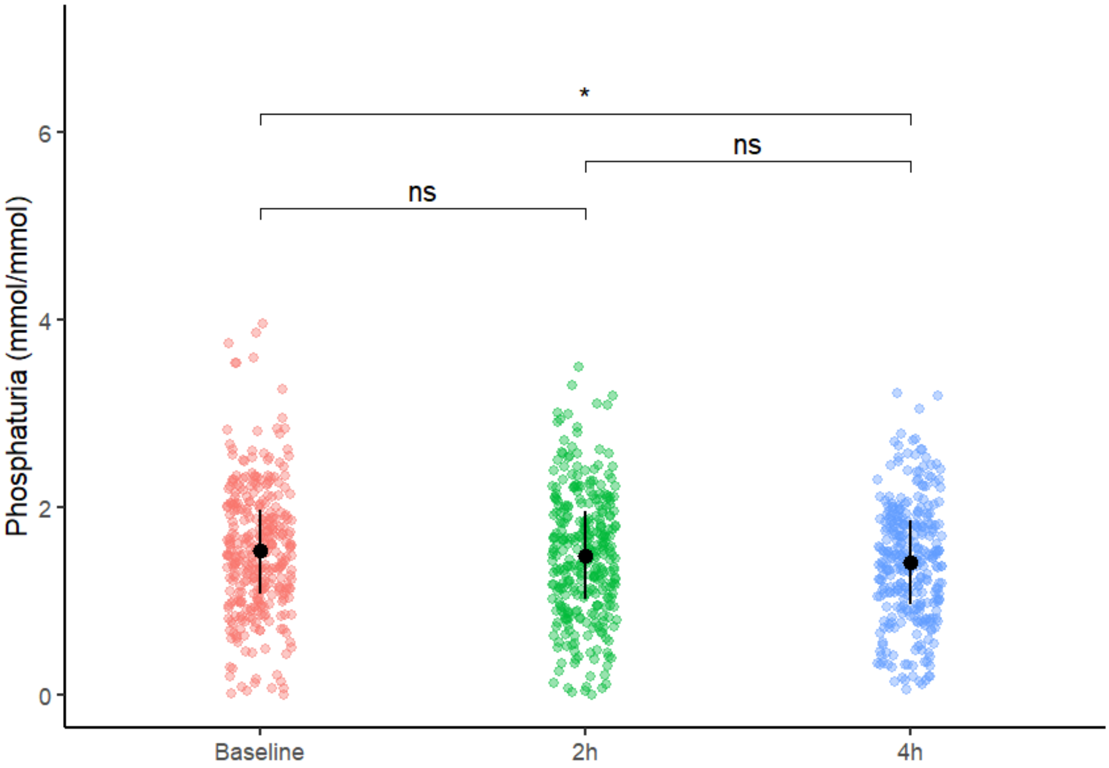

E.

Supplementary Figure 2: Changes of the A. Total serum calcium, B. Ionized serum calcium, C. Parathormone, D. Phosphatemia and E. Urinary phosphate/creatinine ratio across the CLT between baseline, 2h and 4h. A paired Wilcoxon test has been performed for paired comparison. ns = no significant,

*=p<0,01, **=p<0,001, ***=p<0,0001 ****=p <0,00001.

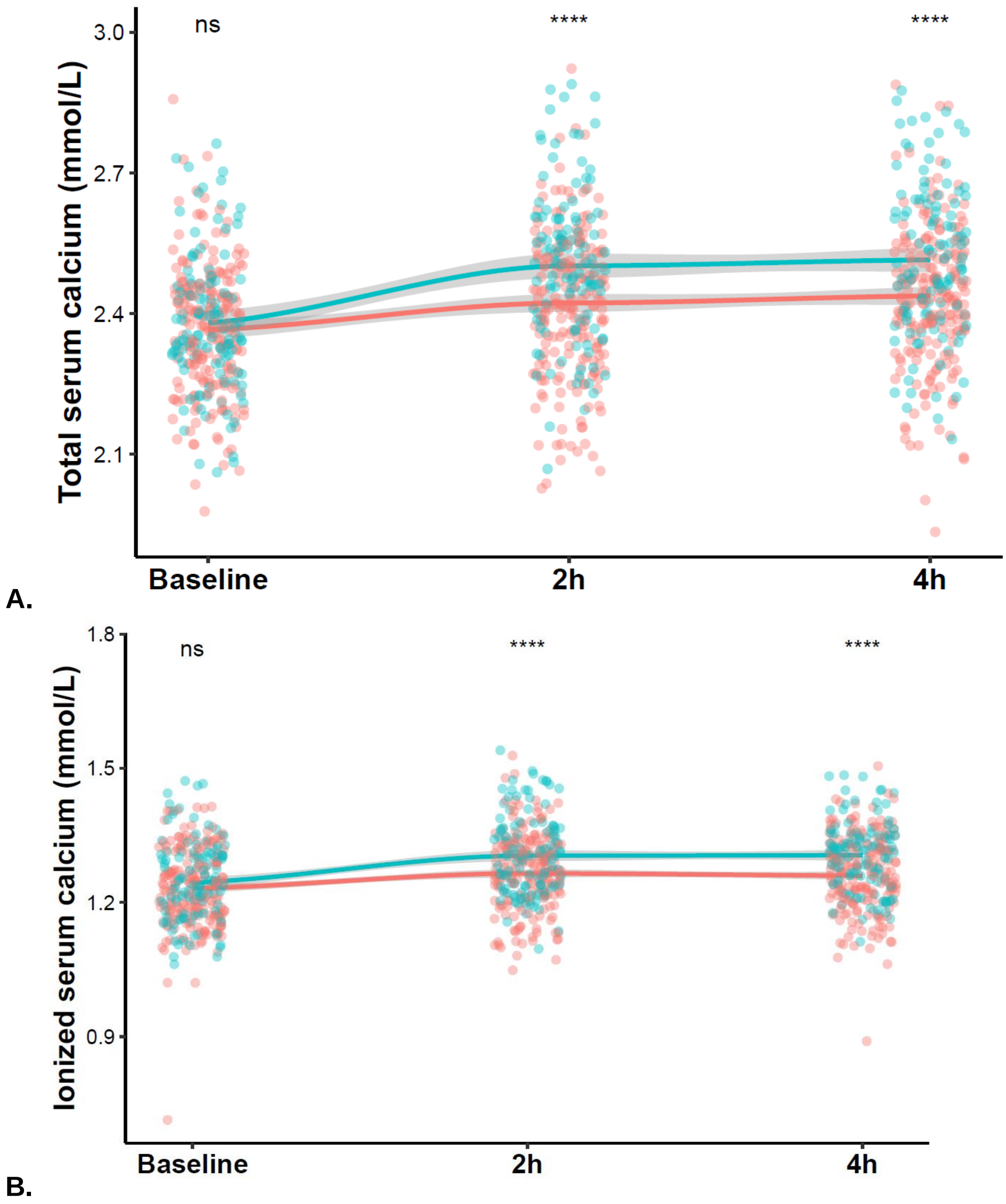
ns
****
****
Total serum calcium (mmol/L)
3.0
2.7
2.4
2.1
Baseline
2h
4h
A.
ns
****
****
Ionized serum calcium (mmol/L)
1.8
1.5
1.2
0.9
Baseline
2h
4h
B.

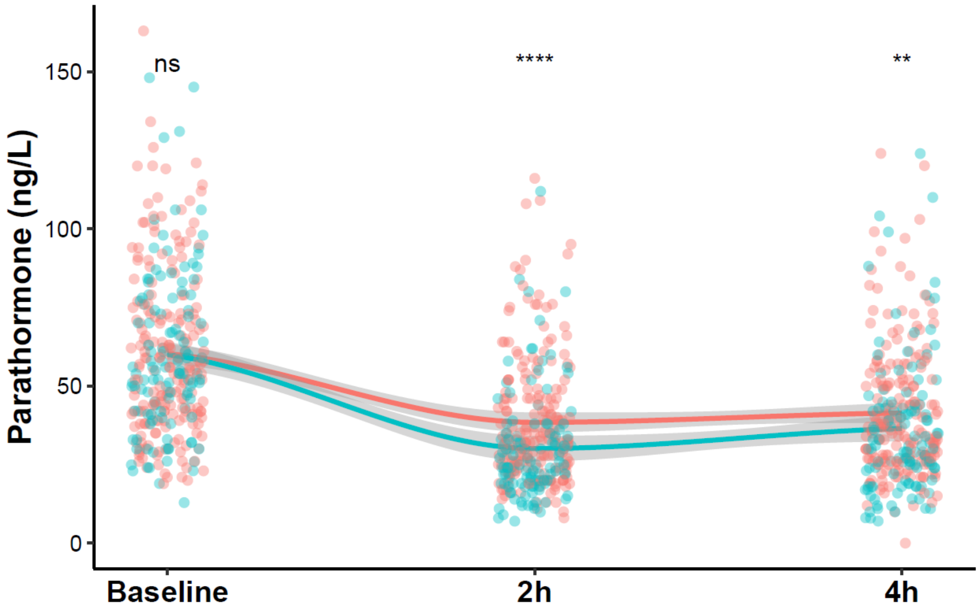

C.

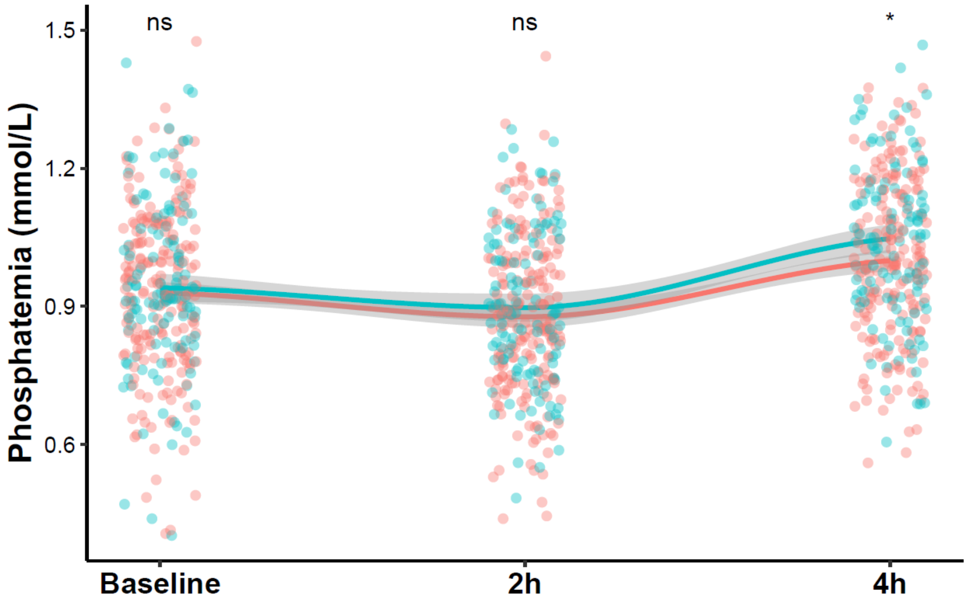

D.

Supplementary Figure 3: Changes of the A. Total serum calcium, B. Ionized serum calcium, C. Parathormone and D. Phosphatemia between baseline, 2h and 4h and in the AH (blue) and no AH (red) group. A paired Wilcoxon test has been performed for paired comparison. ns = no significant, *=p<0,01, **=p<0,001, ***=p<0,0001 ****=p <0,00001.

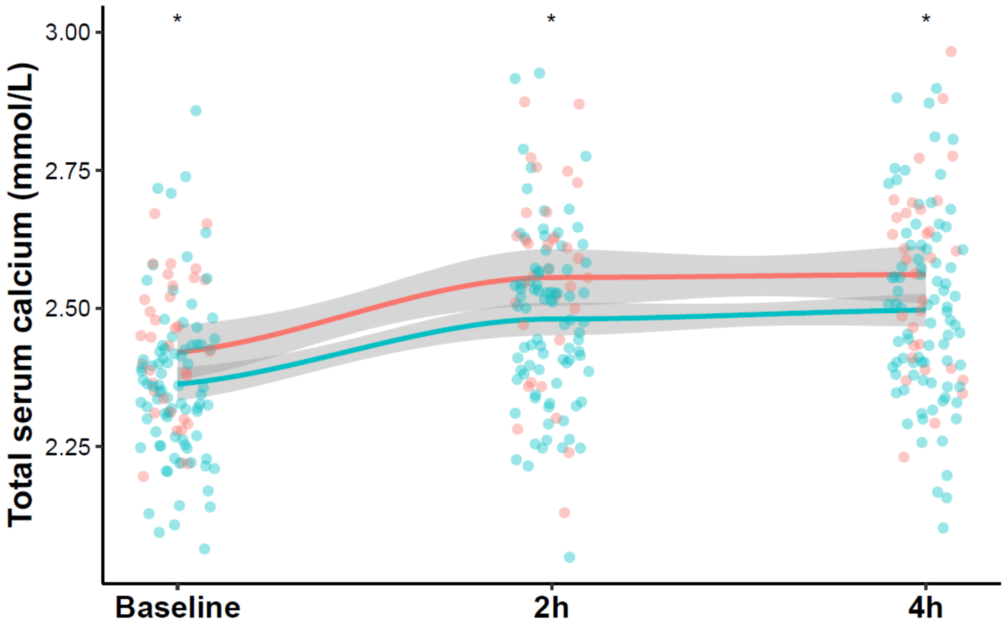
Total serum calcium (mmol/L)
3.00
2.75
2.50
2.25
*
*
*
Baseline
2h
4h

A.

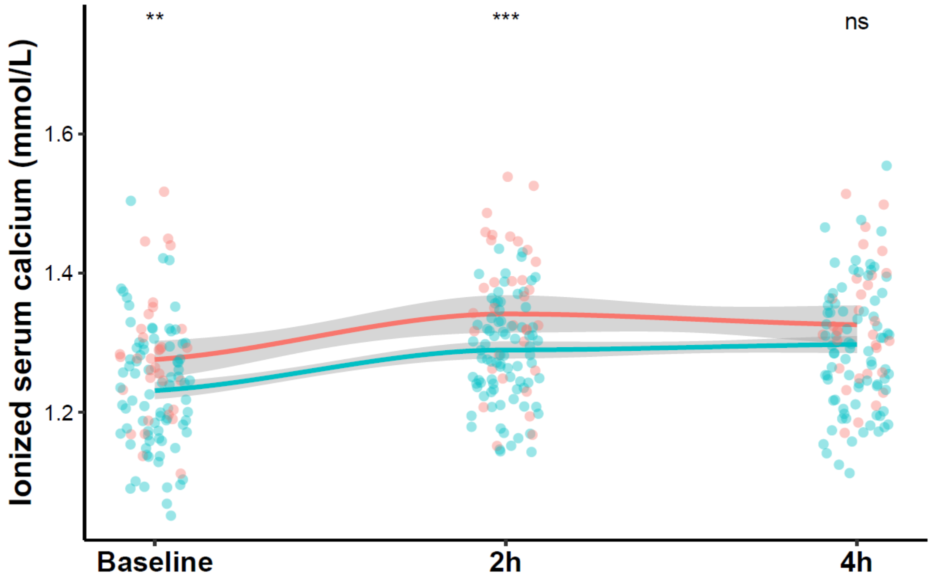
Ionized serum calcium (mmol/L)
1.6
1.4
1.2
**
***
ns
Baseline
2h
4h

B.

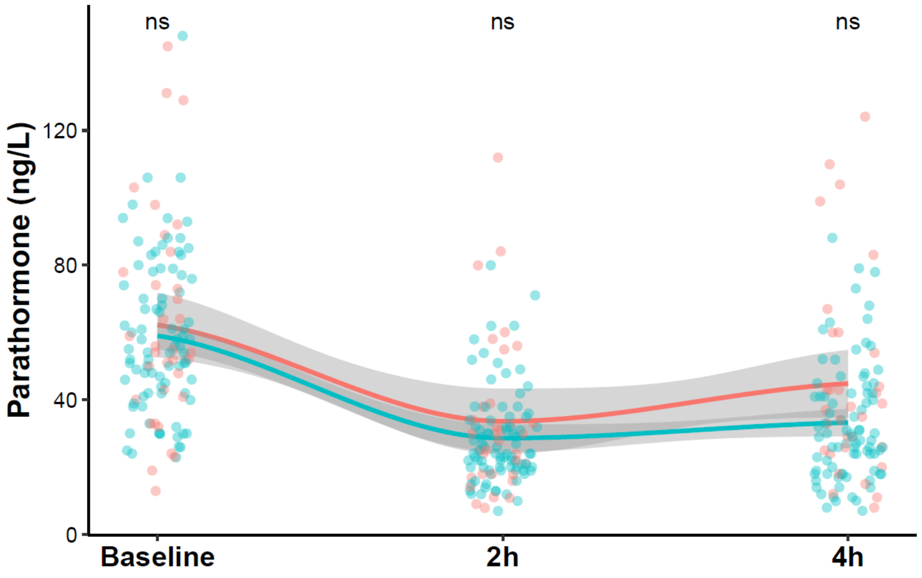

C.

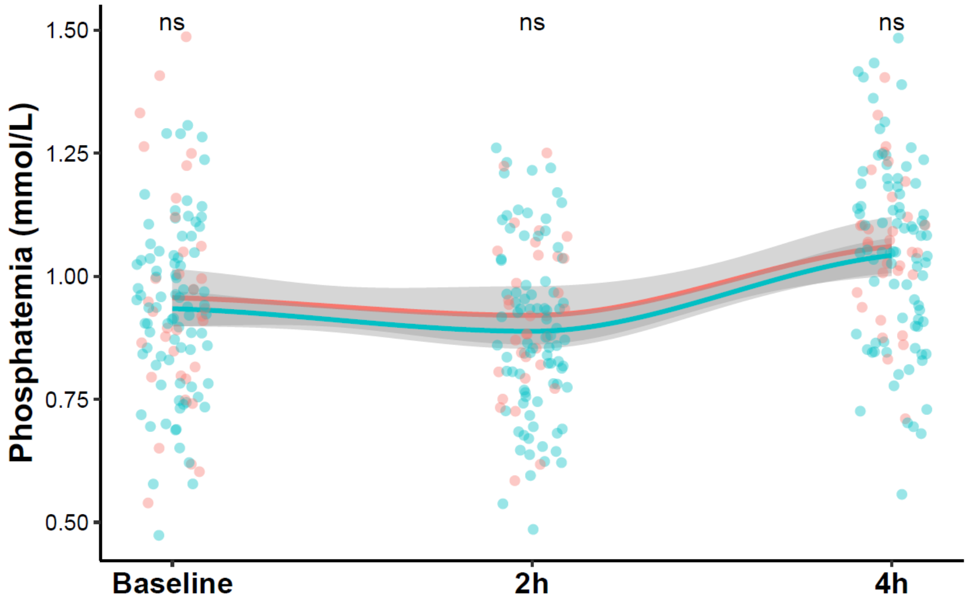

D.

Supplementary Figure 4: Changes of the A. Total serum calcium, B. Ionized serum calcium, C. Parathormone and D. Phosphatemia between baseline, 2h and 4h and in the early AH (red) and late AH (blue) group. A paired Wilcoxon test has been performed for paired comparison. ns = no significant, *=p<0,01, **=p<0,001, ***=p<0,0001 ****=p <0,00001.
